# Spatial analysis to assess the relationship between human and bovine brucellosis in South Korea, 2005–2010

**DOI:** 10.1038/s41598-019-43043-7

**Published:** 2019-04-30

**Authors:** Jun-Sik Lim, Kyung-Duk Min, Sukhyun Ryu, Seung-Sik Hwang, Sung-Il Cho

**Affiliations:** 1 Disease Diagnostic Team, Gyeonggi Veterinary Service Center, Suwon, South Korea; 2 Department of Public Health Science, Graduate School of Public Health, Seoul National University, Seoul, South Korea; 3 WHO Collaborating Centre for Infectious Disease Epidemiology and Control, School of Public Health, Li Ka Shing Faculty of Medicine, The University of Hong Kong, Hong Kong SARS, China

**Keywords:** Policy and public health in microbiology, Bacterial infection, Risk factors

## Abstract

The first case of human brucellosis in South Korea was reported in 2002, and cases of human infection continue to occur. Although an association between human and bovine brucellosis has been identified, the spatial relationship has not been studied in South Korea. Here, we analysed the spatial patterns of human and bovine brucellosis retrieved from the human and veterinary surveillance data, as well as the spatial correlation between human and bovine brucellosis and associated factors that contribute to its occurrence. The risk of human brucellosis was analysed using a Bayesian spatial model with potential risk factors. Our results show that, for both human and bovine brucellosis, hotspots were clustered in the southeast regions of Korea, whereas coldspots were clustered in the northwest regions of Korea. Our study suggests that the risk of human brucellosis increases in rural regions with the highest risk of bovine brucellosis. Collaborative strategies between human and veterinary health sectors (e.g, public health intervention and region-specific eradication programs for bovine brucellosis) would reduce the burden of brucellosis in South Korea.

## Introduction

Brucellosis is a globally neglected zoonotic disease^1^. Human infection with *Brucella* species, known as human brucellosis, occurs through ingestion of contaminated dairy products (e.g., raw milk and cheese); it also occurs through contact with body fluid, aborted foetal tissues, or inhalation of aerosolised animal products. The pathogens of primary concern for public health include *Brucella melitensis* and *Brucella abortus*^2^, the main reservoirs of which are sheep or goat, and cattle, respectively. *B*. *melitensis* is commonly transmitted to humans through a food-borne route; conversely, *B*. *abortus* is generally transmitted through an animal-contact route^3^. Human-to-human transmission of brucellosis is rare, although there have been a few reported cases of infection through breastfeeding, sexual contact, and organ transplantation^4^. Therefore, control of human brucellosis requires policies to prevent and eradicate animal brucellosis^3^. *B*. *melitensis* is the most common zoonotic pathogen worldwide. However, in South Korea, most reported cases of human and animal brucellosis have been caused by *B*. *abortus*; thus, intervention measures in South Korea have been primarily focused on the control of bovine brucellosis^3,5–7^.

Bovine brucellosis has continuously been reported in South Korea since an initial report in 1955, involving dairy cattle imported from the United States^8^. To control the disease, “test and slaughter” programs have been conducted since the 1960s. However, prior to the 2000s, control policy was primarily focused on management of dairy cattle. In early 2004, an intensive eradication program was conducted that involved both dairy and beef cattle^9^. Despite the implementation of control measures, a total of 74,492 cases of bovine brucellosis were reported between January 2005 and December 2010.

The first human case of *B*. *abortus* infection in South Korea was reported in 2002, involving an individual who was infected through consumption of unpasteurized milk^10^. After 2002, the number of human cases continued to increase; the highest number (215 cases) was reported in 2006, and has been followed by a decreasing but continuous rate of infection^11^. To control human brucellosis, investigations of transmission route are needed, including an understanding of the association with bovine brucellosis.

To date, studies of the relationship between the human and bovine brucellosis in South Korea have solely focused on temporal and microbiological aspects^5,6,9,12^; this has limited the establishment of control and prevention strategies. Previous spatial studies of other diseases (e.g., malaria and bovine spongiform encephalopathy) have uncovered the sources of infection, risk populations, and potential transmission routes^13,14^. Spatial analysis of human brucellosis has revealed high-risk areas, associated factors, and routes of transmission from livestock^15,16^. Identification of the spatial patterns of human brucellosis and its associated factors is expected to help policymakers to allocate resources and design region-specific policies; moreover, these data constitute useful information that can support the development of novel intervention methods. In the present study, we analysed the spatial characteristics of human brucellosis and its associated factors, including the risk of bovine brucellosis. Furthermore, we conducted spatial analyses to assess the relationships of these factors with the risk of human brucellosis.

## Methods

### Study design and spatial units

This ecological study involved whole regions of South Korea. During the study period, the number of administrative regions (cities and districts) increased from 250 to 251^17^. All data including disease occurrences and demographics were collected at the administrative district level, from January 2005 to December 2010; address data were transformed using spatial units established in 2010.

### Data source

Incidence data for human brucellosis were obtained from the Infectious Disease Statistics System of the Korea Centers for Disease Control and Prevention^11^. Cases imported from foreign countries were excluded from this analysis. Human population information was collected from the database of the Population and Housing Census in South Korea; this survey is conducted once every 5 years; thus, only population data from 2005 and 2010 were extracted. The median human population number between the two periods was used as a denominator to estimate the incidence of human brucellosis^17^.

Data for bovine brucellosis were extracted from the Animal Health Integrated System of the Animal and Plant Quarantine Agency in South Korea^18^. Data regarding cattle population and breed type (beef cattle, dairy cattle, and mixed breeds raised for beef) were obtained from the database of the Census of Agriculture, Forestry and Fisheries in Statistics Korea; this survey is conducted once every 5 years^17^. The median cattle population number (using 2005 and 2010 data) was used as a denominator to estimate the incidence of bovine brucellosis.

Mean herd size, which is used as a proxy for the magnitude of the farm industry, was obtained from the database of the Census of Agriculture, Forestry and Fisheries in South Korea. Rural population was used as a proxy for rurality^17^. To identify the effect of slaughterhouses where workers experience a risk of contact with the organs of infected animals, the district level addresses of slaughterhouses in 2006 were collected from the Animal and Plant Quarantine Agency in South Korea^19^.

The shapefile of the Korean map in 2010 was obtained from the database of the Statistical Geographic Information Service in Statistics Korea^20^.

### Case definition

Reports of human brucellosis were based on passive surveillance. When infection of the bovine brucellosis was confirmed, epidemiological investigations were conducted for the related individuals. Individuals who had related clinical symptoms and epidemiological characteristics (e.g., contact with cattle) were classified as suspected cases. Among these individuals, any positive results from antibody tests, using the standard tube agglutination test, microagglutination test, polymerase chain reaction for gene or antigen detection, and blood culture led to definitive diagnoses^5,21^. Laboratory diagnosis was conducted at the provincial Public Health Laboratory or Korea Centers for Disease Control and Prevention.

Both active and passive surveillance were performed for bovine brucellosis: screening for bulk raw milk bimonthly; mandatory annual test and pre-trade test for the animals; and upon farmers’ requests for diagnosis. Therefore, diagnostic tests for the disease were conducted upon cattle several times per year in South Korea. For dairy cattle, the milk ring test was conducted for bulk raw milk as a screening test. The Rose Bengal test and plate agglutination test were performed for beef cattle and for dairy cattle that were ≥12 months of age in herds that showed positive results in the milk ring test; these tests were also performed for cattle before they are traded. Confirmatory tests included the enzyme-linked immunosorbent assay, complement fixation test, or tube agglutination test; these were conducted on cattle that showed positive results in the Rose Bengal or plate agglutination tests. If any serological tests were positive, the cattle were recognized as confirmed cases of brucellosis^6^. Bovine cases were diagnosed at the provincial Veterinary Service Center^22^.

### Cluster analysis

Univariate and bivariate cluster analyses were conducted to assess the risks of human and bovine brucellosis. Univariate analyses included univariate Global Moran’s I and local indicator of spatial association (LISA). The Global Moran’s I was conducted to detect whether there was spatial autocorrelation over an entire study area, but not for specific locations. LISA, which is the decomposition of univariate Global Moran’s I into individual regions, was performed to identify local clusters and assess the significance of these clusters^23^. To identify the spatial correlation between the two variables, a bivariate cluster analyses including bivariate Global Moran’s I and bivariate LISA (BiLISA) were performed as the extension of univariate cluster analyses^24^.

Smoothed estimates of standardized incidence ratio (SIR) were used to identify the risks of the diseases in cluster analysis. The raw value of the SIR includes characteristics of uncertainty that depend on the population size: if the size of the population is large, the variance of the SIR is small; otherwise, the variance is large. These problems can be avoided through borrowing of neighbourhood information. In this study, a global empirical Bayes smoothing method was conducted using a Poisson model^25^. This method represents spatial variation, which adjusts for uncertainty. The smoothed estimates are calculated with the pooled mean of the entire study and the weighted local raw value, depending on the number of populations in the region.

To estimate SIR, the incidence rate of human brucellosis per person–year during the study period was calculated as the number of newly reported human cases divided by the person-year at risk. The SIR of bovine brucellosis per cattle–year in the same period was also estimated. In the SIR of bovine cases, adjustment was made for cattle breeds, including beef cattle, dairy cattle, and mixed breeds raised for beef. Regions without cattle were excluded. Adjustment of SIR for human cases was not performed due to the lack of demographic data. SIRs of both diseases were smoothed.

Univariate cluster analyses were conducted for each of the two diseases; bivariate cluster analyses were conducted for the spatial correlations between the risk of human brucellosis and other variables, including the risk of bovine brucellosis, rural population, cattle population, number of slaughterhouses, and mean herd sizes. The row-standardized queen type of first-order contiguity was used to present neighbours. Statistical significance was calculated through Monte Carlo simulation, conducted 999 times with default values. *p-*values less than 5% were considered to be statistically significant.

### Bayesian spatial model

A Bayesian spatial model was developed to examine the effects of certain factors on the risk of human brucellosis. The SIR of the disease was the dependent variable; the smoothed estimates of the SIR of bovine brucellosis, rural population, cattle population, the number of slaughterhouses and mean herd size were selected as independent variables. Mean herd size was modified to a categorical variable based on the median value.

Based on a preliminary study in which the SIRs of human brucellosis were positively skewed with many zeros (38.32%, 82 of 214 regions), a zero-inflated Poisson model was selected. The model and standard Poisson models were non-nested; therefore, the Vuong test was used to compare the fitness of the models^26^. The Vuong test supported the use of a zero-inflated Poisson model, rather than a standard Poisson model (*p* < 0.01). The zero-inflated Poisson model was a mixture of the zero-inflated and Poisson models, which consider two types of zeros: structural zeros and sample zeros. The zero-inflated model was used in regions where the structurally observable value is zero (i.e., there was no at-risk population in the region). The Poisson model was used for observed zeros in regions where other values can be observed.

To include spatial dependence, the Bayesian spatial zero-inflated Poisson model was fitted using integrated nested Laplace approximations (INLA). INLA is an estimation tool for Bayesian analysis, which eases the computational burden but approximates accurate posterior distribution. In INLA, fitting logistic regression for structural zeros is not permitted; therefore, only probabilities can be estimated for structural zeros^27^. The probability function of the number of cases in the Bayesian spatial model can be expressed as^28,29^:

$$p({y}_{i}|{\lambda }_{i},{\pi }_{0})={\pi }_{0}I({y}_{i}=0)+(1-{\pi }_{0})\frac{\exp \,(\,-\,{\lambda }_{i}){{\lambda }_{i}}^{{y}_{i}}}{{y}_{i}!}$$

where $I({y}_{i}=0)$ is the indicator variable; *y*_*i*_ is the observed number of cases; *λ*_*i*_ is the average number of incidences; and *π*_0_ is the probability for structural zero. In cases where *y*_*i*_ is not structural zero, *y*_*i*_ and *λ*_*i*_ can be expressed as^28,30^:

$$\begin{array}{ccc}{y}_{i} &  \sim  & {\rm{Poisson}}({\lambda }_{i})\\ \mathrm{log}({\lambda }_{i}) & = & \alpha +{\beta }_{i}{X}_{i}+{\upsilon }_{i}+{\nu }_{i}+\,\mathrm{log}({E}_{i})\end{array}$$

where *α* is the baseline SIR of human brucellosis; *β*_*i*_ is the regression coefficient of independent variables; *X*_*i*_ is the set of independent variables; ${\upsilon }_{i}$ is the structured spatial random effect for region *i*; ${\nu }_{i}$ is the non-spatial random effect for region *i*; and *E*_*i*_ is the expected number of cases. All prior distributions were selected as *vague* priors^28^, which are default in INLA. The spatial random effect was constructed using an intrinsic conditional autoregressive structure. The non-spatial random effect specified following a Gaussian distribution, with a mean of zero. Regions that had no cattle were excluded from the model. To conduct the univariable Bayesian zero-inflated Poisson model, variables that were significant at the 20% level were selected in the Bayesian spatial model.

In order to ensure the fitness of the Bayesian spatial model for these data, three reduced models for random effect terms (i.e., spatial and non-spatial random effects) were developed to compare the goodness of fit of each model. All models were compared using deviance information criterion (DIC), which is a form of the Akaike information criterion for Bayesian modeling^31^.

Choropleth maps showing the risk of human brucellosis were produced using the posterior mean values of SIRs of human brucellosis, derived from the Bayesian spatial model.

Data management and statistical analyses were conducted using *spdep*^32^ and *INLA* packages^33^ in R software 3.2.4^34^, and GeoDa 1.12^35^.

## Results

### Descriptive results

From January 2005 to December 2010, a total of 540 cases of human brucellosis were reported, excluding one case that was non-domestic in origin. During the same period, a total of 74,492 cases of bovine brucellosis were reported. The incidence rate of human brucellosis in South Korea during the study period was 0.18 cases per 100,000 person–years, and the incidence rate of bovine brucellosis was 0.46 cases per 100 cattle–years. In 2006, there were 73 slaughterhouses nationwide (Table 1). In 38 districts, the SIR of bovine brucellosis was not measured due to the absence of cattle.

**Table 1:** Descriptive statistics.

Variable	Scale	Mean	SD^**^	Minimum	Median	Max
Human population	1 person	204840.00	157982.90	16781.00	174965.00	634941.00
Cattle population	100 cattle	128.65	142.89	0.14	86.90	778.62
SIR^*^ of human brucellosis	—	2.68	5.11	0.00	0.30	29.58
- Smoothed estimates	—	2.25	3.96	0.02	0.35	22.15
SIR^*^ of bovine brucellosis	—	0.90	1.15	0.00	0.56	9.09
- Smoothed estimates	—	0.93	1.06	0.00	0.62	8.87
Mean herd size	1 cattle	34.10	15.93	0.00	31.78	99.85
Rural population	100 people	13.13	14.04	0.00	10.47	86.24
Number of slaughterhouses	1 ea	0.34	0.65	0.00	0.00	5.00

*SIR: Standardized incidence ratio.

**SD: Standard deviation.

### Cluster analyses

Table 2 shows the spatial autocorrelation of the risks of human and bovine brucellosis. The risk of human brucellosis was positively autocorrelated (I = 0.30, *p*-value < 0.01). The risk of bovine brucellosis was also positively autocorrelated (I = 0.22, *p*-value < 0.01).

**Table 2:** Results of univariate and bivariate Moran’s I.

	Variable	I	E(I)	SD(I)	Z	*p*-value
Univariate Global Moran’s I	Human brucellosis	0.30	0.00	0.04	7.83	<0.01
	Bovine brucellosis	0.22	0.00	0.04	5.41	<0.01
Bivariate Global Moran’s I	Bovine brucellosis	0.11	0.00	0.03	3.19	0.01
	Rural population	0.35	0.00	0.03	10.65	<0.01
	Cattle population	0.24	0.00	0.04	6.53	<0.01
	Mean herd size	−0.03	0.00	0.04	−0.77	0.23
	Number of slaughterhouses	0.12	0.00	0.03	3.81	<0.01

**Figure 1:**
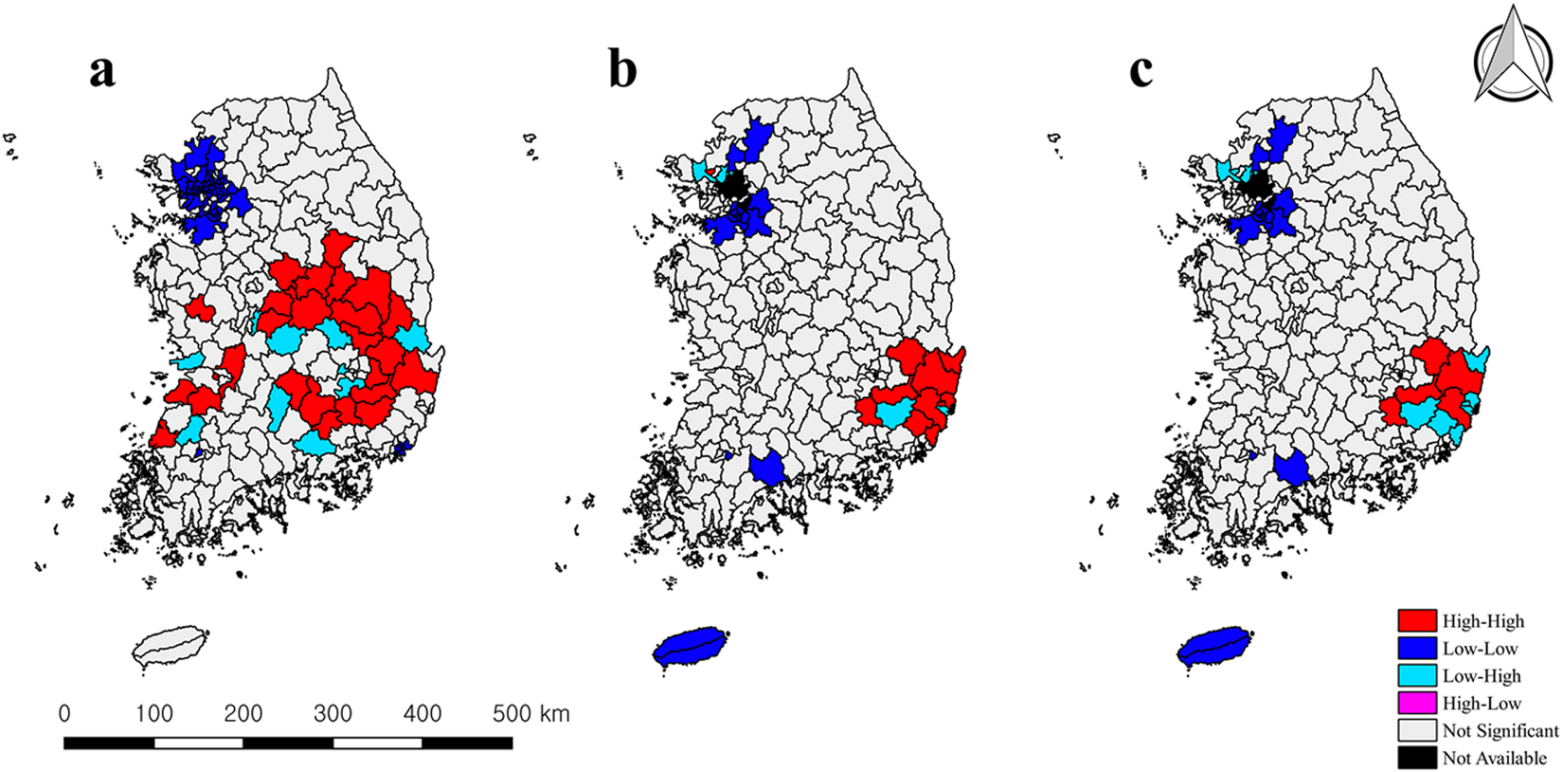
LISA and BiLISA cluster maps of human brucellosis and bovine brucellosis in Korea, 2005–2010 (**a**) LISA of human brucellosis, (**b**) LISA of bovine brucellosis (**c**) BiLISA of human and bovine brucellosis.

The cluster maps for human and bovine brucellosis in Fig. 1 show spatial clusters and their spatial heterogeneities of dependence. “High-High” and “Low-Low” clusters indicated significant positive spatial autocorrelations: these are respective hotspots and coldspots (e.g., a high risk of disease in cases was associated with high risk of disease in neighbours). “High-Low” and “Low-High” clusters indicated negative autocorrelations (e.g., a high risk of disease in cases was associated with low risk of disease in neighbours). Hotspots and coldspots of human cases were located in the southeast/central and northwest regions, respectively (Fig. 1a). Similar to the clusters of human brucellosis, hotspots and coldspots of bovine brucellosis were located in the southeast and northwest regions, respectively. Je-ju Island was identified as a coldspot (Fig. 1b).

Table 2 shows the spatial correlations between the risk of human brucellosis and specific factors. The risk of human brucellosis was positively correlated with the risk of bovine brucellosis (I = 0.11, *p*-value = 0.01), rural population (I = 0.35, *p*-value < 0.01), cattle population (I = 0.2393, *p*-value < 0.01), and number of slaughterhouses (I = 0.12, *p*-value < 0.01). However, the spatial correlation with mean herd size was not statistically significant (I = −0.03, *p*-value = 0.223).

**Figure 2:**
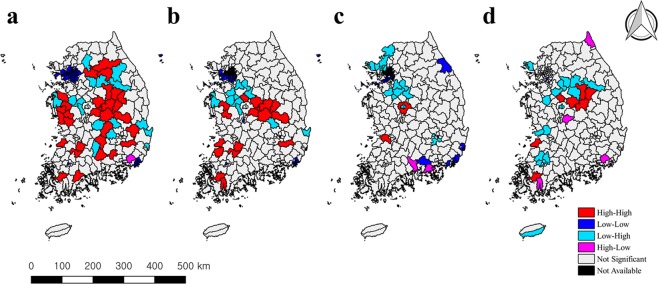
BiLISA cluster maps of risk of human brucellosis and risk factors in Korea, 2005–2010. (**a**) BiLISA cluster map of the risk of HB and rural population. (**b**) BiLISA cluster map of the risk of HB and cattle population. (**c**) BiLISA cluster map of the risk of HB and mean herd size. (**d**) BiLISA cluster map of the risk of HB and the number of slaughtershouse.

Figures 1c and 2 show the cluster patterns of spatial correlations between human brucellosis and specific factors. Each of the five factors showed a different cluster pattern. The risk of human brucellosis had a positive spatial correlation with the risk of bovine brucellosis in the southeast region (hotspot) as well as in the northwest region and Je-ju Island (coldspot) (Fig. 1c). Figure 2a shows the spatial correlation between rural populations and the risk of disease. Positive correlations were located in centre-north and centre-west regions (hotspots), as well as in the northwest region (coldspot) (Fig. 2a). The positive spatial correlations between cattle population and the risk of the disease were located in the centre/cental-west regions (hotspot) and northwest regions (coldspots). Positive correlation patterns between mean herd size and the risk of the disease were identified in few regions (Fig. 2c). With regard to the number of slaughterhouses, hotspots were located in the central regions (Fig. 2d).

### Bayesian spatial model

Estimates of associations in a univariable Bayesian zero-inflated Poisson model are shown in Table 3. Four variables were statistically significant at the 20% level: the risk of bovine brucellosis [risk ratio (RR) = 1.16, 80% credible interval (CI) = 1.11–1.21], rural population (RR = 1.03, 80% CI = 1.03–1.37), cattle population (RR = 1.01, 80% CI = 1.00–1.01), and number of slaughterhouses (RR = 1.24, 80% CI = 1.14–1.35). Levels of correlation and multicollinearity were low among the four variables, which were used in the Bayesian spatial model. The model, which includes both spatial and non-spatial random effects, was the most closely fitted model to this data; it showed the lowest DIC (DIC = 718.93), compared with other reduced random effects models (Table 4).

**Table 3:** Results of univariable analysis using Bayesian zero-inflated Poisson model.

Variable	Risk ratio	80% credible interval
Smoothed SIR^*^ of bovine brucellosis	1.16	1.11–1.21
Rural population	1.03	1.03–1.37
Cattle population	1.01	1.00–1.01
**Mean herd size**		
<31.78	—	—
>31.78	0.89	0.77–1.04
Number of slaughterhouses	1.24	1.14–1.35

*SIR: Standardized incidence ratio.

**Table 4:** Model comparison for the different random effect terms.

Model	Deviance information criterion
Both spatial and non-spatial random effects (Bayesian spatial model)	718.93
Only spatial random effect	810.81
Only non-spatial random effect	818.27
No random effects (Bayesian Zero-inflated Poisson model)	1278.06

Parameter estimates of associations between the risk of human brucellosis and specific factors in the Bayesian spatial model are shown in Table 5. The SIR of human brucellosis showed a significantly positive association with the smoothed estimates of SIR of the bovine brucellosis (RR = 1.49, 95% CI = 1.22–1.82); it also showed a positive association with rural population (RR = 1.04, 95% CI = 1.01–1.07) and cattle population (RR = 1.01, 95% CI = 1.00–1.01). In contrast, the number of slaughterhouses was not significantly associated with SIR of human brucellosis (RR = 0.91, 95% CI = 0.69–1.20).

**Table 5:** Multivariable regression results using Bayesian spatial model.

Variable	Risk ratio	95% credible interval
Smoothed estimates of SIR* of bovine brucellosis	1.49	1.22–1.82
Rural population	1.04	1.01–1.07
Cattle population	1.01	1.00–1.01
Number of slaughterhouses	0.91	0.69–1.20

*SIR: Standardized incidence ratio.

**Figure 3:**
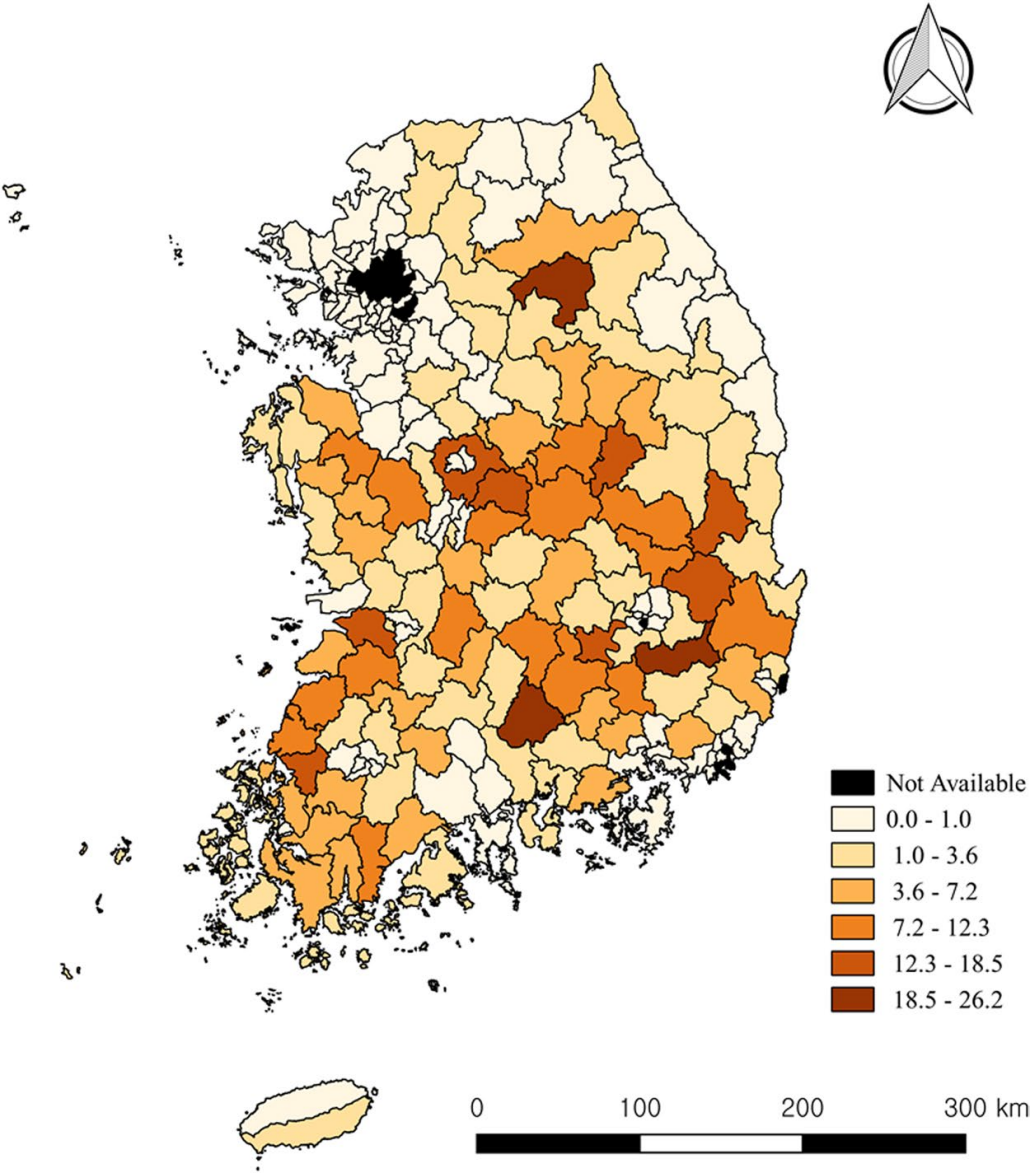
Choropleth map showing posterior mean values of SIR of human brucellosis from Bayesian spatial zero-inflated model.

Risk maps of human brucellosis were represented in Fig. 3.

## Discussion

Understanding the spatial characteristics of a disease aids in determining its aetiology^36^. In the USA, spatial clusters of human brucellosis have been shown to be related to the ethnicities of subsets of the population and their unique food customs, such as eating raw cheese. In China, researchers showed that spatial clusters of human brucellosis were related to livestock^15,16^. Although the relationships between human and bovine brucellosis in South Korea has already been identified^5,6,9,12^, their spatial characteristics are not well-understood. In this study, using data retrieved from human and veterinary surveillance systems, cluster analyses of human and bovine brucellosis were conducted to identify high-risk areas and their spatial correlations. Furthermore, factors associated with human brucellosis were investigated at the district level to suggest intervention methods.

Human brucellosis is not a transmissible disease between humans^2,4,37^; thus, clustering of human cases would indicate common sources of infection^15^, such as animal-contact or food-borne routes. Spatial heterogeneity of dependence in the disease suggests that the risk of human infection might not be related to dairy products in the market; otherwise, spatial variation would not show significant variation. Moreover, in South Korea, pasteurization of dairy products is conducted. Therefore, the occurrence of disease through food purchased on the market might be very low. Considering the contagious characteristics of bovine brucellosis between cattle, districts with many cattle would be at high risk of infection^38^. In this study, identified high-risk districts had prominent cattle industries, which is consistent with previous studies^6,39^.

Spatial correlation and the estimated associations between risks of human and bovine brucellosis suggest that human *Brucella* infection is related to spatial closeness with cattle, either through animal-contact or raw food-borne routes. Therefore, as the cattle population increases, exposures of humans to infected animals or raw dairy products may be more frequent. However, this could also be affected by the extent and proportion of cattle covered by the bovine brucellosis eradication program. The proportion of cattle subject to testing was broadened during the study period^40^. Notably, asymptomatic *Brucella* infections might exist in cattle that were not included in the eradication program; those cattle could have been sources of human infection.

This study revealed that the risk for human brucellosis is high in rural regions where the risk of bovine brucellosis is high. Based on these, the risk map of human brucellosis was produced. Regional interventions for human infection should focus on two sectors: human health and animal health. In South Korea, awareness of brucellosis was high in at-risk populations. However, they did not know prevention methods and felt inconvenienced by wearing personal protective equipment (PPE)^41,42^. Health education for prevention measures should be performed in regions with high risks of brucellosis, because PPE can protect against infection. This can impact not only on brucellosis, but also on other zoonotic diseases^43,44^.

Eradication programs for bovine brucellosis have steadily expanded to increase frequency of testing and the proportion of cattle covered by the programs^22^. Combined with the compensation program for slaughtered cattle infected with *Brucella*, these programs have been successful in controlling the disease^40^. However, these programs have had difficulty in adjusting for the conditions of the individual farms. Risk-level data that we obtained in this study may be helpful for implementing differentiated and more precise eradication programs^6^.

The number of slaughterhouses in a specific area was not significantly related to the risk of human brucellosis. Slaughterhouse workers are a known risk group because they experienced a high possibility of direct contact with animal products, such as organs and body fluids^41,44,45^. This lower risk may be because slaughterhouse workers constitute a small proportion of all infected people, or because of pre-trade testing for cattle as part of the eradication program.

Several previous studies of animal infectious disease, including brucellosis, were conducted using the livestock trade network, with consideration of the ability of the disease to be transmitted between animals^46,47^. Based on the present results showing a spatial relationship between the two diseases, control of zoonotic transmission of brucellosis may also be enhanced through understanding of animal-human contact in the livestock trade network^48^. Further studies incorporating networks for zoonotic infections are recommended.

This study has some limitations. First, as an ecological study, it may incur an element of ecological fallacy when the results are interpreted at the individual level. Second, the spatial unit used in this study was the city or district level. In the Infectious Disease Statistics System of the Korea Centers for Disease Control and Prevention^11^, it was not possible to retrieve demographic data associated with human cases (e.g., age, sex, and occupation). Thus, relationships of these factors could not be investigated. Nevertheless, the effects of rural population and spatial patterns of human and bovine brucellosis indicate that the risk of human infection is strongly associated with agriculture. Moreover, previous studies of human brucellosis in South Korea showed that most cases involved elderly males who were associated with agriculture^5,49^. Third, diagnostic methods for brucellosis have low validity^50,51^. Therefore, some cases of human and bovine brucellosis may have not been identified (i.e., misclassification bias). However, epidemiological investigations combined with the use of multiple diagnostic methods for human cases may reduce the probability of pseudo-negative results. Similarly, the use of multiple diagnostic tests for cattle may reduce the number of undetected cases. Furthermore, according to a previous study^52^, spatial dependence and patterns of disease are not strongly affected by limitations of diagnostic methods, especially for large datasets. Consequently, misclassification bias is not expected to have seriously affected the results. Fourth, the Bayesian models identified a low risk ratio for the smoothed ratio of bovine brucellosis. However, the smoothed ratios of bovine brucellosis were distributed between 0 and 8.87. Thus, the region with the highest risk of bovine brucellosis has approximately 34.8-fold greater risk of human brucellosis than the region with no risk of bovine brucellosis, when other variables are adjusted appropriately. Similarly, risk ratios were 24.68-fold and 10.44-fold greater when the rural and cattle populations were compared between maximum and minimum values, respectively. Additionally, the at-risk population in South Korea is typically a rural population; their proportion of the total population in South Korea is small (Table 1). Therefore, risk ratios indicate that bovine brucellosis and its veterinary policy have considerable implications on the incidence of human brucellosis. Lastly, it is difficult to identify the main route of transmission.

Despite our inability to confirm the main route of transmission, the spatial closeness of the two diseases supports the animal-contact route as the main transmission method in South Korea. First, beef cattle comprise the majority of the cattle population in South Korea^6^. Therefore, the risk of human infection might be primarily related to *Brucella* infection of beef cattle. A possible route of infection from beef cattle is through contact with raw meat products. However, the likelihood of this route is considered minimal because meat products seldom transmit *Brucella* species^53^. Second, nearly all cases of human brucellosis in South Korea were *B*. *abortus* infections, and the occupational characteristics and history of patients supported transmission through the animal-contact route^5,49^. Taken together, these facts indicate that zoonotic transmission of brucellosis in South Korea primarily occurs through animal contact. However, considering that there have been a few reports of food-borne infection^53^, that route cannot be underestimated.

To our knowledge, this was the first study to analyse the spatial relationship between human and bovine brucellosis in South Korea. Human brucellosis was significantly spatially clustered with bovine brucellosis. Animal-level intervention for zoonosis led to benefits for human health^54^; therefore, a stricter eradication program for bovine brucellosis is needed in rural regions with a high risk of cattle infection combined with the information of risk map of human brucellosis. Moreover, health policies, such as health education for epidemiology of brucellosis and associated prevention, should be implemented in rural regions. Collaborative approaches with human and veterinary health are needed to control brucellosis^55,56^. We expect that the data from this study will aid in implementing veterinary and public health policy.

## Data Availability

The datasets generated and analysed during the current study are available from the corresponding author on reasonable request.
